# The Influence of Moderate Physical Activity on Brain Monoaminergic Responses to Binge-Patterned Alcohol Ingestion in Female Mice

**DOI:** 10.3389/fnbeh.2021.639790

**Published:** 2021-02-25

**Authors:** Trevor J. Buhr, Carter H. Reed, Allyse Shoeman, Ella E. Bauer, Rudy J. Valentine, Peter J. Clark

**Affiliations:** ^1^Department of Food Science and Human Nutrition, Iowa State University, Ames, IA, United States; ^2^Neuroscience Program, Iowa State University, Ames, IA, United States; ^3^Interdepartmental Graduate Program in Nutritional Sciences, Iowa State University, Ames, IA, United States; ^4^Department of Kinesiology, Iowa State University, Ames, IA, United States

**Keywords:** alcohol abuse, drinking in the dark, exercise, physical activity, monoamine, rodent models, binge drinking, voluntary ethanol consumption

## Abstract

Monoamine neurotransmitter activity in brain reward, limbic, and motor areas play key roles in the motivation to misuse alcohol and can become modified by exercise in a manner that may affect alcohol craving. This study investigated the influence of daily moderate physical activity on monoamine-related neurochemical concentrations across the mouse brain in response to high volume ethanol ingestion. Adult female C57BL/6J mice were housed with or without 2.5 h of daily access to running wheels for 30 days. On the last 5 days, mice participated in the voluntary binge-like ethanol drinking procedure, “Drinking in the dark” (DID). Mice were sampled immediately following the final episode of DID. Monoamine-related neurochemical concentrations were measured across brain regions comprising reward, limbic, and motor circuits using ultra High-Performance Liquid Chromatography (UHPLC). The results suggest that physical activity status did not influence ethanol ingestion during DID. Moreover, daily running wheel access only mildly influenced alcohol-related norepinephrine concentrations in the hypothalamus and prefrontal cortex, as well as serotonin turnover in the hippocampus. However, access to alcohol during DID eliminated wheel running-related decreases of norepinephrine, serotonin, and 5-HIAA content in the hypothalamus, but also to a lesser extent for norepinephrine in the hippocampus and caudal cortical areas. Finally, alcohol access increased serotonin and dopamine-related neurochemical turnover in the striatum and brainstem areas, regardless of physical activity status. Together, these data provide a relatively thorough assessment of monoamine-related neurochemical levels across the brain in response to voluntary binge-patterned ethanol drinking, but also adds to a growing body of research questioning the utility of moderate physical activity as an intervention to curb alcohol abuse.

## Introduction

Alcohol abuse can cause debilitating health issues, despite being amongst the top preventable contributors to worldwide death. Alcohol abuse has also been linked to over 200 diseases including drug dependence, hypertension, type 2 diabetes, and dementia. Thus, there is a need to identify the neurophysiological underpinnings of alcohol abuse to develop effective interventions that curtail its consequences. Evidence indicates that regularly engaging in moderate physical activity may have tremendous therapeutic value for substance use disorders, and therefore may be a beneficial intervention to curb alcohol abuse (Goodwin, 2003). Indeed, individuals that regularly exercise are less likely to engage in substance abuse or experience relapse during recovery from drug dependence (Goodwin, 2003; Smith and Lynch, 2012). Moreover, mounting evidence from rodent models of drug abuse also suggests that exercise therapy may aid in recovery from cocaine, morphine, and methamphetamine dependence (Cosgrove et al., 2002; Miladi-Gorji et al., 2012; Engelmann et al., 2014). However, the impact of physical activity status on alcohol abuse, in particular, remains less clear, as reports with human subjects and rodent models have demonstrated varied outcomes (Giesen et al., 2015; Leasure et al., 2015; Manthou et al., 2016; Horrell et al., 2020). Yet still, regular exercise can promote adaptations in mood regulation and reward neuropathways that also overlap with the motivation to ingest excessive amounts of alcohol (Werme et al., 2002b; Greenwood et al., 2011; Herrera et al., 2016; Robison et al., 2018). Therefore, the possibility remains that these exercise-induced adaptations may influence the motivation to misuse alcohol.

Considerable evidence suggests that the activity of monoamine neurotransmitters, including serotonin (5-HT), dopamine (DA), and norepinephrine (NE) in brain reward, motor, and limbic systems, play key roles in the motivation to drink alcohol (Camarini et al., 2010; Fitzgerald, 2013; Belmer et al., 2016). Therefore, interventions that change the activity of monoamine neurotransmitters may hold therapeutic value for alcohol abuse. Acute bouts of physical activity can stimulate transient 5-HT, DA, and NE activity in brain reward and limbic system structures (Freed and Yamamoto, 1985; Bailey et al., 1992; Dunn et al., 1996; Gomez-Merino et al., 2001; Lin and Kuo, 2013). Furthermore, long-term exercise produces adaptations to the availability of receptors that can modulate the release of monoamines, like the serotonin 1A receptor of the raphe nuclei and dopamine 2 receptor in the striatum (Gilliam et al., 1984; MacRae et al., 1987; Greenwood et al., 2003; Clark et al., 2014; Bauer et al., 2020). Such exercise-induced adaptations may change monoamine neurotransmitter responses during alcohol ingestion, thereby altering the risk for its misuse. However, whether or not physical activity status can influence the activity of monoamines during alcohol ingestion remains unknown.

“Drinking in the dark” (DID) is a rodent model of binge-like ethanol drinking that has contributed greatly to our understanding of the physiological underpinnings of alcohol abuse (Sprow and Thiele, 2012). The National Institute on Alcohol Abuse and Alcoholism defines alcohol binge drinking as a pattern of ethanol ingestion that produces blood alcohol concentrations (BAC) greater than 0.08%. C57BL/6J mice consume high amounts of ethanol under the DID paradigm, reliably reaching BACs greater than 0.1% within a 2-to-4-h period of ethanol access. This results in behavioral signs of intoxication including ataxia and anxiolysis (Rhodes et al., 2005, 2007; Barkley-Levenson and Crabbe, 2015). Thus, the DID model is not only an excellent resource to identify correlates of changes to brain monoaminergic activity that are associated with motivation to consume high levels of alcohol, but also to understand how physical activity status may influence monoamine responses to episodes of binge-like drinking. However, brain monoaminergic activity during voluntary ethanol drinking to binge-like levels has not been comprehensively investigated using rodent models. Furthermore, only a few studies have investigated the interaction between physical activity status and motivation to ingest alcohol in animal models, while typically examining alcohol preference using a two-bottle choice, instead of binge-like drinking (Werme et al., 2002a; Pichard et al., 2009; Darlington et al., 2014; Booher et al., 2019). Thus, there is a need for a more comprehensive examination of how monoaminergic activity becomes influenced during voluntary binge-like alcohol drinking, and how physical activity status might alter such responses. Identifying the effects of binge-like ethanol drinking on the levels of monoamines and metabolites, as well as how physical activity may interact with such responses could provide insights into new approaches that reduce the risk of alcohol abuse.

The purpose of the current study was to investigate the influence of physical activity status on mouse brain monoaminergic responses to binge-like ethanol drinking using the DID paradigm. Brain area-specific markers of monoaminergic activity were investigated immediately following access to ethanol after five consecutive days of DID in mice that were or were not granted daily running wheel access. Monoamines were analyzed by measuring concentrations of the neurotransmitters 5-HT, DA, and NE, along with the DA metabolites 3,4-dihydroxyphenylacetic acid (DOPAC) and homovanillic acid (HVA), 5-HT metabolite 5-hydroxyindoleacetic acid (5-HIAA), and DA pre-cursor levodopa (L-DOPA) using Ultra-High-Performance Liquid Chromatography (UHPLC). Monoamine concentrations were assessed in the prefrontal cortex, hypothalamus, striatum, hippocampus, brainstem, and caudal cortical region, as these areas comprise limbic, reward, and motor pathways that contribute to the misuse of alcohol and cognitive dysfunction related alcohol abuse (Koob, 2014). The results of this study not only provide a relatively thorough assessment of monoamine-related neurochemical changes across the brain in response to voluntary binge-patterned ethanol drinking but may also have implications for the utility of moderate physical activity as an intervention to curb alcohol abuse.

## Materials and Methods

### Rodent Housing

Six-week-old female C57BL/6J mice (Jackson Laboratory) were individually housed upon arrival at standard laboratory conditions with *ad libitum* access to food and water, except during DID sessions, in which 20% ethanol replaced water in the cages where mice were assigned to the alcohol drinking condition. Female mice were chosen for this study because previous work has suggested they drink more ethanol and run further on wheels than male mice (Lightfoot et al., 2004; Rhodes et al., 2007), which we hypothesized would result in a greater likelihood of detecting effect sizes sufficient to observe differences in the dependent measures between groups. Mice were given a 1-week acclimation period to adjust to a reversed light-dark cycle before experimental procedures (described below). All procedures were in accordance with the *Guide for the Care and Use of Laboratory Animals*, 8th Edition (Institute for Laboratory Animal Research, The National Academies Press, Washington, DC, USA, 2011) and were approved by the Iowa State University Institutional Animal Care and Use Committee. All possible efforts were made to minimize the number of animals used and their suffering.

### Wheel Running Paradigm

Mice were randomly placed in one of four groups: no access to running wheels with water during DID (sed/water; *n* = 9), no access to running wheels with alcohol during DID (sed/alcohol; *n* = 9), daily running wheel access with water during DID (run/water; *n* = 9), or daily running wheel access with alcohol during DID (run/alcohol; *n* = 9). Alcohol was only present during a short period following wheel access on the last 5 days (see “Drinking in the Dark” section). At the start of the dark cycle, mice designated for running wheel access were moved from their home cage to a cage with a 4-inch diameter voluntary running wheel for 2.5 h, to encourage moderate physical activity. Wheel rotations were recorded *via* Starr Life Sciences VitalView software. Sedentary mice were also moved from their home cage to a temporary standard cage during this period to control for the change in environment and handling. Mice were returned to their home cages after 2.5 h. This process was repeated daily for approximately 35 days.

### Drinking in the Dark

On day 30, a DID protocol was administered to alcohol-consuming groups for alcohol self-administration over 5 days (Rhodes et al., 2005). After the 2.5-h running period, mice were returned to their home cages and left undisturbed for 30 min before the start of the DID procedure. For DID, water bottles were removed and replaced with 10 ml graduated sipper tubes containing 20% ethanol or water. Mice were allowed to freely consume 20% ethanol for 2 h each day for 4 days and 4 h on the fifth and final day. Four hours of DID was performed on the final day because we hypothesized that longer access to alcohol would yield more drinking and a greater likelihood of observing changes to neurochemical concentrations (Rhodes et al., 2007). The volume of fluid in sipper tubes was recorded every 30 min during DID. Mice were sacrificed *via* rapid decapitation. Brain extraction and dissection occurred immediately following the final DID session. Brain areas containing the prefrontal cortex, remaining caudal cortical area, hypothalamus, cerebellum, striatum, hippocampus, and caudal brainstem areas were rapidly microdissected on a glass plate placed over ice. Microdissection of specific brain areas was completed as follows and considered the stereotaxic coordinates detailed in Paxinos and Franklin’s “Mouse Brain in Stereotaxic Coordinates fifth edition.” Using a flat edge razor blade the olfactory bulbs were removed and disposed of followed by a coronal cut approximately 1.97 mm rostral to bregma to excise the prefrontal cortex area. Vascular tissue (i.e., The circle of Willis) surrounding the hypothalamus area was removed using forceps before extraction of the hypothalamus between approximately 0.13 mm and −2.69 mm anteroposterior to bregma. At approximately 0.83 mm caudal to bregma a razor blade was used to separate the striatum from the remaining brain tissue. The cortical area surrounding the striatum was carefully removed and placed into a vial for caudal cortical tissue. The cerebellum was severed from its point of attachment to the rest of the brainstem using forceps and collected. A mid-sagittal cut through the remaining brain tissue then allowed for access to the hippocampal area which was then collected (excluding cortical tissue) from each hemisphere. The brainstem area was then separated from the remaining cortical tissue at approximately −2.69 mm, −8.15 mm to bregma. The remaining cortical tissue was then added to the vial for caudal cortical tissue. Microdissected brain areas were placed in pre-weighed cryovials containing 0.2 M perchloric acid, flash-frozen with liquid nitrogen, weighed again to obtain sample weights (see Supplementary Table 2.1), and then stored at −80°C in an Ultra-Low freezer until UHPLC processing.

### Western Blot Analysis

The amount of protein synthesis enzyme, phosphorylated p70S6K1, was measured in the gastrocnemius muscle of all mice, as a marker of muscle adaptation to running wheel access (Drummond et al., 2009). Immediately after decapitation, the gastrocnemius was extracted and flash-frozen with liquid nitrogen then stored at −80°C until the time of analysis. Western blot analysis followed previously described methods (Valentine et al., 2018). Approximately 15–25 μg of protein were separated by 4–15% gradient Stain-Free Criterion TGX gel electrophoresis (Bio-Rad, Hercules, CA, USA), and transferred onto a polyvinylidene difluoride membrane (MilliporeSigma, Burlington, MA, USA). Gels were activated according to Bio-Rad’s Stain-Free protocol, and total protein was quantified to normalize signal intensity for the protein of interest in each lane to the total protein. The membrane was then blocked in Tris-buffered saline (pH 7.5) containing 0.1% Tween-20 (TBST) and 5% non-fat dry milk for 1 h at room temperature. Next, strips were incubated overnight in primary antibodies at a 1:2,000 dilution. The primary antibodies were directed against p70S6K1 (#2708, Cell Signaling Technology, Danvers, MA, USA) and phospho-p70S6K1 (#9234, Cell Signaling Technology, Danvers, MA, USA), an enzyme involved in the synthesis of proteins related to training adaptations in the muscle. Membranes were then washed with TBST, incubated in horseradish peroxidase-conjugated secondary antibodies against rabbit (Cell Signaling Technology, Danvers, MA, USA) at a 1:5,000 dilution, and washed with TBST and Nano-pure water. The membranes were imaged using an enhanced chemiluminescence solution. Bands were captured with the ChemiDoc™ XRS Imaging System (Bio-Rad, Hercules, CA, USA), and densitometry was performed using Image Lab V6.0 (Bio-Rad, Hercules, CA, USA).

### High-Performance Liquid Chromatography

Immediately following dissection, brain tissue samples were preserved in 0.2 M HCLO4 and stored at −80°C until UHPLC preparation. Brain regions were homogenized using the Omni Bead Ruptor system. Sample homogenates were then centrifuged for 30 min at 3,000 *g*. An Ultra-High-Performance Liquid Chromatography with electrochemical detection (UHPLC-ECD) system was used to determine neurochemical and metabolite concentrations as described previously (Villageliú et al., 2018). A Dionex Ultimate 3000 autosampler system queued samples before injection. Separation of neurochemical target compounds was completed *via* a 150 mm long Hypersil BDS C18 column (Thermo Scientific, Sunnyvale, CA, USA) using 0.6 ml min^−1^ flow rate of 10% buffered acetonitrile MD-TM mobile phase (Thermo Scientific, Sunnyvale, CA, USA). The column used had a diameter of 3 mm. Particle and pore size was 3 μm and 130 Å, respectively. Electrochemical detection was achieved with 6041RS glassy carbon electrode at 400 mV with a limit of detection set at 5× the signal-to-noise ratio. Neurochemical standards were purchased from Sigma–Aldrich (St. Louis, MO, USA).

### Statistical Analysis

Statistical analysis was performed using SAS Enterprise Guide 7.1 and GraphPad Prism 8.2.0. A repeated-measures ANOVA was performed on daily running distance with week, access to running wheels, and access to alcohol during DID as factors. A repeated-measures ANOVA was also performed on daily ethanol intake over each session of DID procedures. Change in body mass during the study (i.e., week 1 to week 5) and phosphorylated p70SK1 levels in the gastrocnemius muscle was analyzed using a two-way ANOVA, with access to wheels and alcohol during DID as factors.

Neurochemical measurements obtained from UHPLC were corrected for gram weight of total tissue (i.e., μg of neurochemical/gram of tissue). The ratio of metabolite to respective neurotransmitter concentration [i.e., 5-HIAA/5-HT, (HVA + DOPAC)/DA) was also calculated as a measure of neurotransmitter turnover (Nissbrandt and Carlsson, 1987; Phillips et al., 1989)]. Two-way ANOVAs were performed to compare neurochemical concentrations or turnover in each brain area with exercise (sedentary vs. running) and DID treatment (water vs. alcohol) as factors. All ANOVAs with statistically significant interactions or main effects for both factors (i.e., sedentary vs. running, and water vs. alcohol) were followed by *post hoc* analyses using pairwise *t*-tests with Fisher’s least significant difference corrections for multiple comparisons. Neurochemical concentrations that were greater than two standard deviations from the mean were excluded from analysis, as noted in the degrees of freedom reported for ANOVAs.

For neurochemical data, the results were only reported for statistically significant values to conserve space. Group means (±SEM) and test statistics for all neurochemicals across each brain region can be located in Supplementary Tables 1.1–1.9.

## Results

### Wheel Running, Body Mass Change, and Associated Muscle Adaption

**Figure 1:**
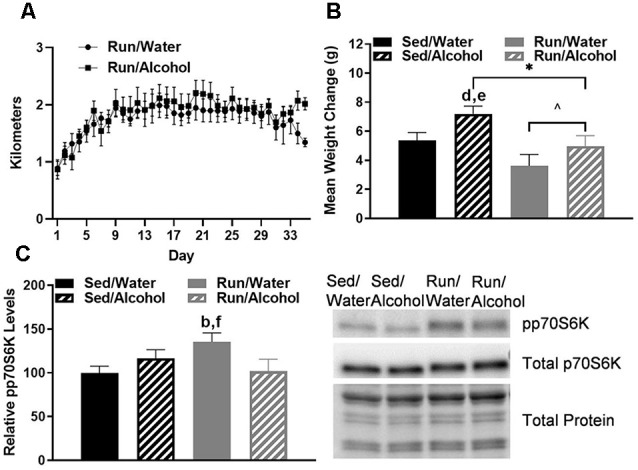
Daily running distance and related phosphorylated p70S6K marker for muscle protein synthesis. **(A)** Average daily running distances (±SEM) for mice throughout the study. **(B)** Average weight change throughout the study, and **(C)** western blot analysis containing the relative optical density for phosphorylated p70S6K corrected for total protein in mouse gastrocnemius muscle (left) and representative blot images (right). *P < 0.05 main effect alcohol access, ^∧^*P* < 0.05 main effect physical activity status, ^b^*P* < 0.05 Sed/Water from Run/Water, ^d^*P* < 0.05 Sed/Alcohol from Run/Water, ^e^*P* < 0.05 Sed/Alcohol from Run/Alcohol, ^f^*P* < 0.05 Run/Water from Run/Alcohol. Data represent +SEM.

The average daily running distance gradually increased from 0.88 km and plateaued at approximately 1.8 km per day (±SEM = 0.05). Throughout the entire study, the average distance ran for mice with access to water was 1.75 km (±SEM = 0.03), whereas mice with access to alcohol ran 1.85 km per day (±SEM = 0.04; see Figure 1A). Alcohol access (*F*_(1,32)_ = 5.76, *p* = 0.022) and running wheel access (*F*_(1,32)_ = 8.98, *p* = 0.005) influenced the amount of mass gained by mice throughout the study (see Figure 1B). *Post hoc* analysis revealed that sedentary mice with access to alcohol gained more mass than running mice with access to water (*p* = 0.0006) or ethanol (*p* = 0.02).

Finally, mice with wheel access had an increase of phosphorylated p70S6K1 in the gastrocnemius as compared to mice that did not receive access to alcohol (*F*_(1,30)_ = 5.49, *p* = 0.026; see Figure 1C). Indeed, the *post hoc* analysis revealed that wheel running mice with access to water during DID had greater muscle p70S6K1 protein density than sedentary mice with access to water (*p* = 0.026) and wheel running mice with access to ethanol (*p* = 0.034).

### Drinking Data

**Figure 2:**
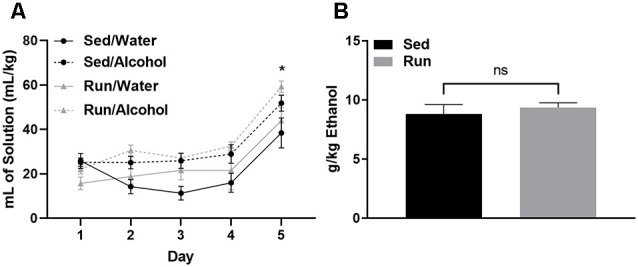
Ethanol consumption during the drinking in the dark protocol. Comparison between the average amount of fluid ingested (±SEM) over the 5-day DID procedure (relative to body mass) for mice that had access to alcohol and water **(A)** and total ethanol intake (relative to body mass) during the final DID session **(B)**. *P < 0.05 Main effect of day, ^ns^*P* > 0.05 between Run/Alcohol and Sed/Alcohol. Data represent means +SEM.

Physical activity status did not influence the amount of fluid ingested relative to bodyweight for either mice that had access to water or alcohol (*F*_(1,32)_ = 0.00, *p* = 0.94; see Figure 2A). However, mice with access to ethanol consumed greater volumes of fluid compared to those with access to water during DID (*F*_(3,32)_ = 7.293, *p* < 0.0007). Mice consumed almost double the volume of ethanol or water on day 5 during the 4-h DID session, when compared to the first 4 days that consisted of 2-h DID sessions (*F*_(4,123)_ = 32.26, *p* < 0.0001; see Figure 2A). The amount of ethanol ingested on day 5 of DID was 8.82 g/kg (±SEM = 0.8) for sedentary mice and 9.36 g/kg (±SEM = 0.4) for mice with access to running wheels (see Figure 2B). Finally, wheel access did not influence the amounts of fluids ingested independent of fluid type (*F*_(1,32)_ = 2.87, *p* = 0.11), or while considering day and fluid type (*F*_(4,123)_ = 0.52, *p* = 0.72).

### Striatum Area

**Figure 3:**
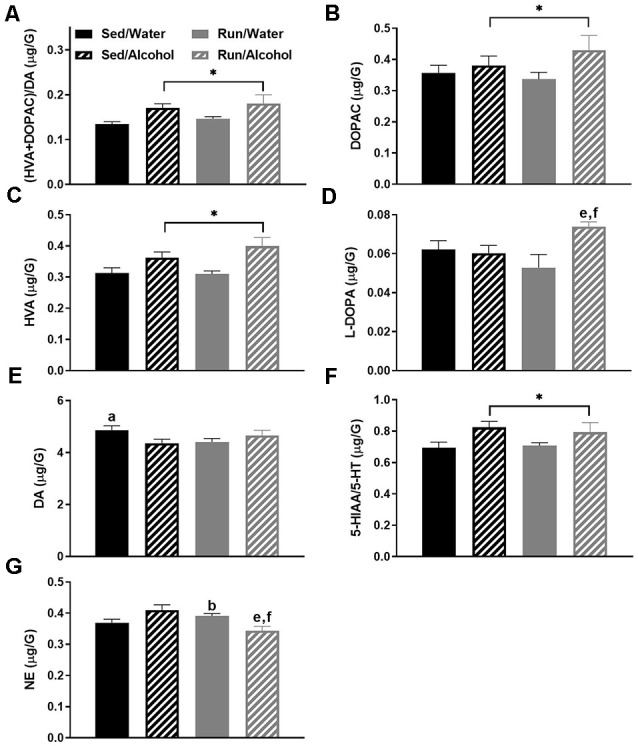
Neurochemical levels in the mouse striatum area immediately following the final DID session. Ratio of (HVA + DOPAC)/DA **(A)**, DOPAC concentrations **(B)**, HVA concentrations **(C)**, L-DOPA concentrations **(D)**, DA concentrations **(E)**, ratio of 5-HIAA/5-HT **(F)**, NE concentrations **(G)**. Data represent means +SEM. *P < 0.05 main effect alcohol access, ^a^*P* < 0.05 Sed/Water from Sed/Alcohol, ^b^*P* < 0.05 Sed/Water from Run/Water, ^e^*P* < 0.05 Sed/Alcohol from Run/Alcohol, ^f^*P* < 0.05 Run/Water from Run/Alcohol.

The striatal area had the most statistically significant changes to neurochemical concentrations, which were primarily a result of alcohol access during DID. Ethanol ingestion robustly influenced markers of DA-related neurochemical activity. Indeed, mice that received access to ethanol had a greater (DOPAC + HVA)/DA ratio (*F*_(1,32)_ = 9.86, *p* < 0.0036) in the striatum, suggesting an overall increase in DA turnover as a result of alcohol ingestion (see Figure 3A). With regards to individual neurochemicals, access to ethanol during the DID period increased the levels of DA metabolites, DOPAC (*F*_(1,32)_ = 4.22, *p* < 0.05) and HVA (*F*_(1,32)_ = 13.53, *p* < 0.0009; see Figures 3B,C). Running and access to alcohol interacted to change L-DOPA (*F*_(1,28)_ = 4.30, *p* < 0.0475) and DA (*F*_(1,32)_ = 5.33, *p* = 0.0276) concentrations in the striatum. *Post hoc* analysis revealed that running mice with access to ethanol had greater L-DOPA than their sedentary mice counterparts (*p* = 0.0365), as well as running mice that had access to water (*p* = 0.0023; see Figure 3D). Furthermore, DA content was lower in sedentary mice that received alcohol compared to those that had water during DID (*p* = 0.0377; see Figure 3E).

In addition to changes in DA related neurochemicals, mice that received access to ethanol had an increased 5-HIAA/5-HT ratio (*F*_(1,32)_ = 7.01, *p* = 0.0125), suggesting alcohol ingestion also augmented 5-HT turnover in the striatum (see Figure 3F). Finally, a statistically significant interaction was observed between alcohol and exercise conditions for NE concentrations (*F*_(1,32)_ = 10.69, *p* = 0.0026), whereby *post hoc* analysis revealed running mice with alcohol access had lower NE compared to running mice with water access (*p* = 0.0014) and sedentary mice with ethanol access (*p* = 0.04). Moreover, for mice that had access to water, running increased NE concentrations in the striatum compared to the sedentary condition (*p* = 0.0170; see Figure 3G).

### Hypothalamic Area

**Figure 4:**
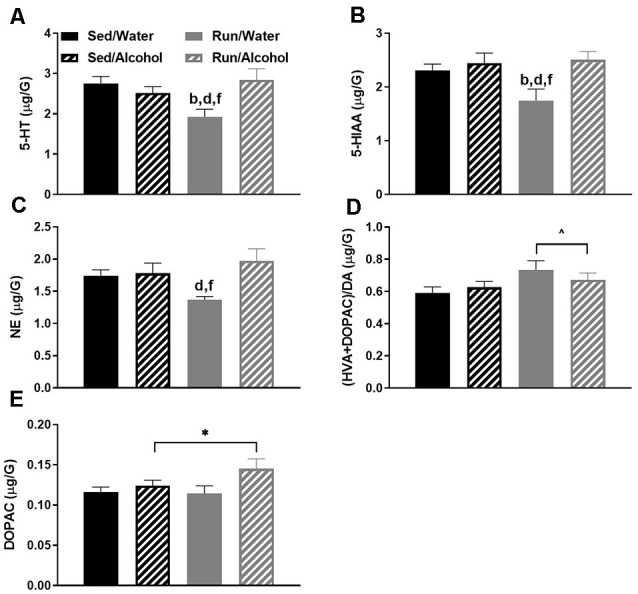
Neurochemical levels in the mouse hypothalamus area immediately following the final DID session. 5-HT concentrations **(A)**, 5-HIAA concentrations **(B)**, NE concentrations **(C)**, ratio of (HVA + DOPAC)/DA **(D)**, and DOPAC concentrations **(E)**. Animals that ran and were exposed to DID had increased DOPAC levels **(E)**. Data represent means +SEM. *P < 0.05 main effect alcohol access, ^∧^*P* < 0.05 main effect physical activity status, ^b^*P* < 0.05 Sed/Water from Run/Water, ^d^*P* < 0.05 Sed/Alcohol from Run/Water, ^f^*P* < 0.05 Run/Water from Run/Alcohol.

The hypothalamus had five statistically significant neurochemical changes. The most striking of which was observed with the interactions between physical activity status and ethanol access for hypothalamic 5-HT (*F*_(1,32)_ = 7.97, *p* = 0.0081), 5-HIAA (*F*_(3,32)_ = 4.05, *p* = 0.0152), and NE (*F*_(1,32)_ = 4.18, *p* = 0.0436) content. Overall, access to ethanol eliminated exercise-induced reductions of 5-HT, 5-HIAA, and NE concentrations in the hypothalamus. Indeed, running mice with access to water had lower 5-HT concentrations than sedentary mice with access to water (*p* = 0.0071) or alcohol (*p* = 0.0481), as well as running mice with access to alcohol (*p* = 0.0033; see Figure 4A). Paralleling 5-HT levels, running mice with access to water also had lower 5-HIAA concentrations than their ethanol drinking counterparts (*p* = 0.0038), as well as sedentary mice that had access to water (*p* = 0.0272) and ethanol (*p* = 0.0074; see Figure 4B). Finally, running mice with access to water also had lower NE concentrations than their ethanol drinking counterparts did (*p* = 0.0031), as well as sedentary mice that had access to ethanol (*p* = 0.0375; see Figure 4C). A statistically non-significant trend between sedentary and running mice with access to water was observed for NE (*p* = 0.0609).

Additionally, some changes reflecting DA turnover were observed within the hypothalamus. Running increased the ratio of (DOPAC + HVA)/DA in the hypothalamus compared to sedentary mice, independent of ethanol access (*F*_(1,32)_ = 4.68, *p* = 0.0386; see Figure 4D). Moreover, ethanol access increased hypothalamic DOPAC concentrations independent of physical activity status (*F*_(1,32)_ = 4.75, *p* = 0.0367; see Figure 4E).

### Brainstem Area

**Figure 5:**
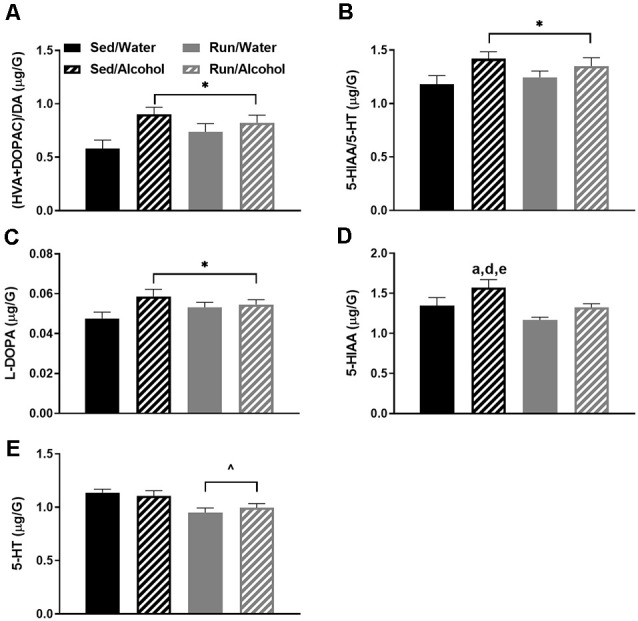
Neurochemical levels in the mouse brainstem area immediately following the final DID session. The ratio of (HVA + DOPAC)/DA **(A)**, the ratio of 5-HIAA/5-HT **(B)**, L-DOPA concentrations **(C)**, 5-HIAA concentrations **(D)**, and 5-HT concentrations **(E)**. Data represent means +SEM. *P < 0.05 main effect alcohol access, ^∧^*P* < 0.05 main effect physical activity status, ^a^*P* < 0.05 Sed/Water from Sed/Alcohol, ^d^*P* < 0.05 Sed/Water from Run/Water, ^e^*P* < 0.05 Sed/Alcohol from Run/Alcohol.

The brainstem area also had five statistically significant changes in monoamine-related neurochemicals. Most notably, ethanol ingestion increased the ratios of both (DOPAC + HVA)/DA (*F*_(1,32)_ = 7.92, *p* = 0.0096; see Figure 5A) and 5-HIAA/5-HT (*F*_(1,32)_ = 5.79, *p* = 0.0220; see Figure 5B), which may suggest that alcohol drinking augments turnover of these neurotransmitters systems at their areas of cell body origin (Pickel et al., 1975; Steinbusch et al., 1979). With regards to specific neurochemical changes, L-DOPA concentrations were also mildly increased in mice that had access to ethanol (*F*_(1,32)_ = 4.47, *p* = 0.0423; see Figure 5C). Physical activity status (*F*_(1,32)_ = 7.82, *p* = 0.0087) and ethanol access (*F*_(1,32)_ = 6.27, *p* = 0.0176) influenced 5-HIAA levels in brainstem regions, whereby sedentary mice with access to ethanol had higher concentrations of 5-HIAA than sedentary mice with access to water (*p* = 0.0280) and running mice with access to ethanol (*p* = 0.0442) or water (*p* = 0.0007; see Figure 5D). Finally, exercise lowered 5-HT concentrations in the brainstem regions, independent of ethanol access during the DID period (*F*_(1,32)_ = 13.25, *p* = 0.0010; see Figure 5E).

### Caudal Cortical Area

**Figure 6:**
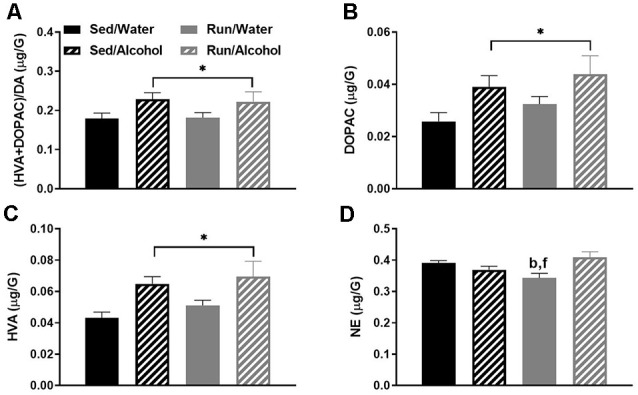
Neurochemical levels in the mouse caudal cortical area immediately following the final DID session. Ratio of (HVA + DOPAC)/DA **(A)**, DOPAC concentrations **(B)**, HVA concentrations **(C)**, and NE concentrations **(D)**. Data represent means +SEM. *P < 0.05 main effect alcohol access, ^b^*P* < 0.05 Sed/Water from Run/Water, ^f^*P* < 0.05 Run/Water from Run/Alcohol.

The remaining cortical area (i.e., with the prefrontal cortex removed) had the next highest number of statistically significant monoaminergic-related changes at four. Of particular interest, access to ethanol during the DID period increased the (DOPAC + HVA)/DA ratio (*F*_(1,32)_ = 6.31, *p* = 0.0173; see Figure 6A). This finding was likely due to the capacity of ethanol access to increase both DA metabolites DOPAC (*F*_(1,32)_ = 6.99, *p* = 0.0126) and HVA (*F*_(1,32)_ = 11.75, *p* = 0.0017), without resulting in changes to DA concentrations (see Figures 6B,C). Moreover, physical activity status and access to alcohol interacted to influence NE concentrations (*F*_(1,32)_ = 7.78, *p* = 0.0088), whereby alcohol access eliminated exercise-induced decreases of NE content (see Figure 6D). Indeed, running mice with access to water during DID had lower NE concentrations than sedentary mice with access to water (*p* = 0.0451) and running mice with access to alcohol (*p* = 0.0113).

#### Cerebellum

**Figure 7:**
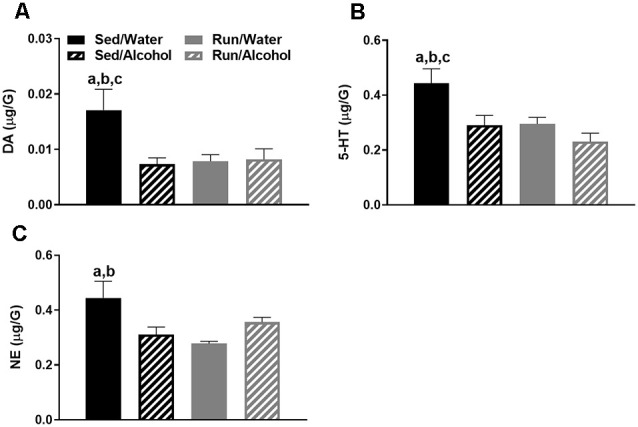
Neurochemical levels in the mouse cerebellum area immediately following the final DID session. DA concentrations **(A)**, 5-HT concentrations **(B)**, and NE concentrations **(C)**. Data represent means +SEM. ^a^*P* < 0.05 Sed/Water from Sed/Alcohol, ^b^*P* < 0.05 Sed/Water from Run/Water, ^c^*P* < 0.05 Sed/Water from Run/Alcohol.

The cerebellum also had four statistically significant changes. Overall, physical activity status and alcohol access interacted to influence DA, 5-HT, and NE related neurochemicals in the cerebellum. Foremost, a statistically significant interaction was found between exercise condition and access to alcohol for DA concentrations (*F*_(1,32)_ = 4.69, *p* = 0.0379; see Figure 7A). Sedentary mice that had access to water had greater amounts of DA compared to their ethanol available counterparts (*p* = 0.0049), as well as running mice that had access to water (*p* = 0.0069) and ethanol (*p* = 0.0076). For cerebellar 5-HT concentrations, statistically significant main effects were observed for both physical activity status (*F*_(1,32)_ = 5.90, *p* = 0.0209) and alcohol access (*F*_(1,32)_ = 6.22, *p* = 0.0180; see Figure 7B). Sedentary mice that had access to water had greater concentrations of 5-HT compared to their ethanol available counterparts (*p* = 0.0076), as well as running mice that had access to water (*p* = 0.0086) and ethanol (*p* = 0.0015). Finally, physical activity status and alcohol access interacted to affect cerebellar NE content (*F*_(1,32)_ = 9.49, *p* = 0.0042; see Figure 7C). Sedentary mice that had access to water had greater concentrations of NE compared to their running (*p* = 0.0017) and ethanol drinking counterparts (*p* = 0.0097).

### Hippocampal Area

**Figure 8:**
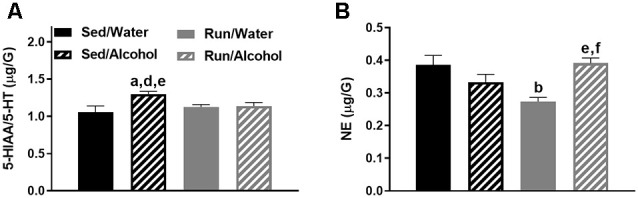
Neurochemical levels in the mouse hippocampus area immediately following the final DID session. The ratio of 5-HIAA/5-HT **(A)**, and NE concentrations **(B)**. Data represent means +SEM. ^a^*P* < 0.05 Sed/Water from Sed/Alcohol, ^b^*P* < 0.05 Sed/Water from Run/Water, ^d^*P* < 0.05 Sed/Alcohol from Run/Water, ^e^*P* < 0.05 Sed/Alcohol from Run/Alcohol, ^f^*P* < 0.05 Run/Water from Run/Alcohol.

In the hippocampus, the interaction between physical activity status and access to ethanol affected the ratio of 5-HIAA/5-HT (*F*_(1,32)_ = 4.51, *p* = 0.0414) and NE content (*F*_(1,32)_ = 16.69, *p* = 0.0003; see Figures 8A,B). Exercise eliminated the increased turnover of hippocampal 5-HT resulting from ethanol access, as sedentary mice with access to ethanol had a greater 5-HIAA/5-HT ratio than running mice with access to ethanol (*p* = 0.0436) or water (*p* = 0.0305), and sedentary mice with access to water (*p* = 0.0034). Finally, access to alcohol eliminated the exercise-induced reductions of hippocampal NE content, as running mice with access to water had lower concentrations of NE than their sedentary (*p* = 0.0008) and alcohol drinking (*p* = 0.0003) counterparts. Running mice with access to ethanol also had marginally greater NE concentrations than their sedentary counterparts did (*p* = 0.0467).

### Prefrontal Cortex

**Figure 9:**
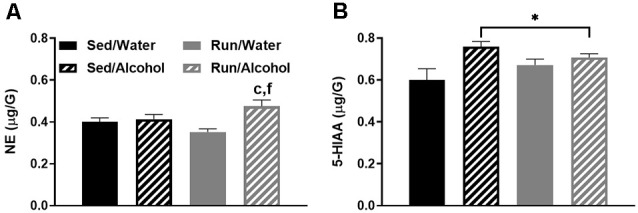
Neurochemical levels in the mouse prefrontal cortex area immediately following the final DID session. NE concentrations **(A)**, and 5-HIAA concentrations **(B)**. Data represent means +SEM. ^*^P < 0.05 main effect alcohol access, ^c^*P* < 0.05 Sed/Water from Run/Alcohol, ^f^*P* < 0.05 Run/Water from Run/Alcohol.

Alcohol access and running interacted to augment NE concentrations in the prefrontal cortex (*F*_(1,32)_ = 6.06, *p* = 0.0194), as running mice with access to ethanol had greater NE levels than both sedentary (*p* = 0.0244) and running mice (*p* = 0.0005) that had access to water (see Figure 9A). Moreover, access to ethanol increased concentrations of 5-HIAA in the prefrontal cortex (*F*_(1,32)_ = 8.21, *p* = 0.0073; see Figure 9B).

## Discussion

This study examined the impact of moderate physical activity on monoamine-related neurochemical responses to binge-like ethanol drinking across mouse brain areas that comprise reward, limbic, and motor systems. The results provided several key findings with regards to the impact of physical activity status and alcohol ingestion on monoamine levels throughout the brain. First, physical activity status did not influence drinking behavior during DID and only mildly affected monoamine-related neurochemical concentrations following alcohol ingestion. In particular, NE levels were slightly elevated in the prefrontal cortex and decreased in the striatum of physically active mice following alcohol access (see Figures 9A, 3G). Moreover, wheel access spared alcohol-related increases of 5-HT turnover markers in the hippocampus (see Figure 8A). Second, alcohol ingestion eliminated some of the exercise-induced changes to monoamines and metabolite concentrations, including reduced NE content in the hypothalamus, hippocampus, and caudal cortical areas, as well as reduced hypothalamic concentrations of 5-HT and 5-HIAA (see Figures 4C, 8B, 6D). Finally, the DID paradigm replicated several key monoaminergic concentration changes, yet also yielded some departures from past work that employed commonly used methods of forced ethanol exposure (e.g., i.p. injection, gavage, vapor chamber, etc.). Some of the parallels between the DID model and previous work included augmented correlates of DA-related activity in the striatal and brainstem areas, as well as greater markers of 5-HT turnover in striatal, hippocampal, and brainstem regions (Fadda et al., 1980; Kiianmaa and Tabakoff, 1983; Hellevuo and Kiianmaa, 1988; see Figures 3, 5, 8). However, alcohol access during DID had a relatively limited influence on neurochemical levels in the prefrontal cortex and in NE concentrations across brain areas, which contrasts some findings of previous work using models of acute ethanol exposure (Pohorecky and Jaffe, 1975; Murphy et al., 1988; Milio and Hadfield, 1992; Lanteri et al., 2008). Overall, these results provide some insight into the potential utility of moderate physical activity to influence neurochemical levels related to the motivation to ingest high volumes of ethanol.

Perhaps the most unexpected outcome of this study was that physical activity status failed to robustly influence monoamine-related neurochemical responses to ethanol ingestion. This outcome was surprising because running can potently modulate monoaminergic activity and receptor plasticity throughout limbic and reward structures, which in turn can alter monoamine responses during distinct events, like exposure to stress or some drugs of abuse (Foley and Fleshner, 2008; Lin and Kuo, 2013; Clark et al., 2015; Arnold et al., 2020; Katsidoni et al., 2020). One possibility is that the relatively mild influence that alcohol ingestion had on monoamine and metabolite concentrations could have limited the ability to detect neurochemical changes that were further influenced by physical activity in this study. Moreover, the possibility remains that despite reaching a daily running distance of nearly 2 km, which was sufficient to elevate markers of muscle adaptation related to training (see Figure 1C), restricting the running period to 2.5 h may not have been enough to maximally stimulate brain adaptations that influence monoaminergic activity in response to binge-like ethanol drinking (Drummond et al., 2009). It should be noted that p70S6K1 protein augmentation related to wheel running was reduced in mice with ethanol access (see Figure 1C), which is consistent with previous work suggesting impaired muscle growth following periods of high-volume alcohol exposure (Lang et al., 2000; Steiner and Lang, 2014, 2015). However, providing longer periods of exercise, just prior to alcohol access may be problematic, as wheel running is rewarding to rodents and the stress associated with suddenly restricting wheel access may result in greater ethanol ingestion (Greenwood et al., 2011, 2012; Nishijima et al., 2013; Herrera et al., 2016). The methods of the current study were designed to minimize alcohol-seeking due to potential stress from restricted wheel access, by providing the same period running every single day in a different environment from the mouse home cages. Moreover, limiting daily wheel access to a couple of hours may more closely model human patterns of engagement in physical activity than continuous wheel access paradigms, where C57BL/6J mice commonly run over the entirety of 12-h periods and can reach distances of 4–9 km per night (Lightfoot et al., 2004; Clark et al., 2011). Therefore, shorter periods of wheel access may be particularly relevant for modeling the potential influence of physical activity status on human physiological responses to alcohol abuse. Taken together, the results of the current study add to a growing, but conflicting body of literature questioning the utility of exercise as a therapeutic approach to mitigate the severity of alcohol abuse disorders.

Nonetheless, mice with daily access to running wheels displayed some differences in neurochemical concentrations in response to alcohol ingestion compared to their sedentary counterparts that could have implications for motivation to misuse alcohol. Daily running mildly increased NE concentrations in the prefrontal cortex, while lowering NE concentrations in the striatum, in response to ethanol access (see Figures 3, 9). Access to running wheels also prevented the elevated markers of 5-HT turnover in the hippocampus that were related to ethanol drinking (see Figure 8). Growing evidence suggests that increases or decreases of brain region-specific noradrenergic signaling are common responses to drugs of abuse, including alcohol (Fitzgerald, 2013; Koob and Volkow, 2016). However, mixed results have been found concerning NE concentrations following acute alcohol exposure in rodent models (Vazey et al., 2018). Moreover, research into the potential alcohol-induced changes to NE in the prefrontal cortex or striatum that have been linked to behavioral or neurophysiological outcomes is limited (Vazey et al., 2018). Some evidence suggests that alcohol’s rewarding properties may depend on heightened NE levels in the prefrontal cortex (Ventura et al., 2006). Therefore, elevated NE levels in the prefrontal cortex could contribute to the urge to drink alcohol that has been reported to follow bouts of exercise (Manthou et al., 2016). However, the current results did not demonstrate an increase in ethanol ingestion in running mice with respect to their sedentary counterparts. Compared to the prefrontal cortex and limbic regions, even less work has been done examining striatal NE levels following acute exposure to ethanol. However, it is worth noting, that a prior history of stress exposure may sensitize striatal NE responses to ethanol exposure (Karkhanis et al., 2014). On the other hand, regularly engaging in physical activity promotes stress-resistance and may (Dishman et al., 1997; Greenwood et al., 2013), desensitize striatal NE activity in response to ethanol. However, the influence of lower striatal NE concentrations on motivation to ingest alcohol remains unresolved. Therefore, more research is required to determine the behavioral or cognitive effects of possible exercise-induced changes to NE levels in the prefrontal cortex and the striatum in response to ethanol ingestion.

Exercise also eliminated the augmented turnover of 5-HT in the hippocampus of mice that had access to ethanol (see Figure 8A). This finding is particularly interesting because augmented 5-HT activity in the hippocampus following exposure to large amounts of ethanol has been linked to impaired hippocampal function that may result from an increased inhibition of principal neurons (McBride et al., 1990; Lovinger, 1997; Bare et al., 1998; Belmer et al., 2018). The capacity of moderate physical activity to attenuate alcohol-increased 5-HT turnover in the hippocampus suggests that regularly engaging in physical activity might protect against some hippocampal dysfunction that has been reported with acute episodes of binge drinking. Extensive research has shown robust improvements to hippocampal function following long periods of regular engagement in physical activity (Van Praag et al., 1999; Christie et al., 2008; Clark et al., 2008; Marlatt et al., 2012; Merritt and Rhodes, 2015). Moreover, regularly participating in exercise can facilitate neuroplasticity (e.g., synapse makers and neurogenesis) in models of chronic alcohol-induced hippocampal deterioration (Redila et al., 2006; Helfer et al., 2009; Hamilton et al., 2012; Maynard and Leasure, 2013). Therefore, the benefits of exercise on hippocampal function may also extend to mitigate short-term or spatial memory deficits during acute alcohol challenges. However, to the best of our knowledge, no studies have investigated the influence of physical activity status on hippocampal function during episodes of binge-like alcohol drinking.

Access to alcohol during the DID period eliminated some of the exercise-induced changes to monoaminergic-related neurochemical concentrations. This was particularly pronounced in the hypothalamic area of wheel running mice, where ethanol ingestion eliminated the reduced concentrations of NE, 5-HT, and 5-HIAA (see Figures 4A–C), but also to a lesser extent in the hippocampal and caudal cortical areas for NE content (see Figures 8B, 6D). A popular view remains in the scientific community that the anxiolytic or antidepressant properties of exercise may depend on elevated 5-HT, DA, and NE concentrations across brain areas comprising reward and limbic circuits (Meeusen et al., 2001). This assertion appears to be primarily based on data measuring monoamine levels during exhaustive exercise when brain monoaminergic activity is high. However, this is less clear when measured during post-exercise periods or rest, as some evidence suggests that 5-HT and NE concentrations may be lower in trained rodent brain areas including the cortex, hypothalamus, and hippocampus, when compared to non-trained conditions (Barchas and Freedman, 1963; Dey et al., 1992; Hoffmann et al., 1994; Gerin and Privat, 1998; Lambert and Jonsdottir, 1998; Gomez-Merino et al., 2001). The capacity of moderate exercise to potentially lower NE- and 5-HT-related neurochemical levels during post-exercise periods in brain regions involved in mediating mood and stress responses, albeit negated by alcohol ingestion in the current study, might still have significant implications for therapeutic approaches targeted at alcohol dependence. Indeed, increased levels of NE in the brainstem, amygdala, and hypothalamus reported during alcohol withdrawal can induce stress-related negative feelings (e.g., anxiety), which may encourage further alcohol-seeking (Trzaskowska and Kostowski, 1983; Walker et al., 2008; Lee et al., 2011; Koob, 2014). The capacity of post-exercise periods to lower basal NE concentrations in brain regions that mediate responses to stress (see Figures 4C, 8B) might suggest regular exercise could be a useful approach to counteract alcohol craving during withdrawal. Indeed, recent work indicates that mice with access to running wheels display attenuated anxiety-like behavior during periods of alcohol withdrawal (Kolb et al., 2013; Motaghinejad et al., 2015; Lynch et al., 2019). The contribution of potential post-exercise attenuated NE or 5-HT concentrations to the management of alcohol craving during withdrawal could be topics for future investigations.

The DID paradigm has many advantages as a model of binge-like ethanol ingestion. Possibly foremost, the DID procedure capitalizes on rodent’s self-motivation to ingest high amounts of ethanol in relatively short periods of time. This is a departure from decades of previous work into the acute impact of alcohol abuse on monoamine levels using rodents, which commonly employed methods of forced high-volume ethanol exposure, involving unconventional stressful routes of alcohol administration (e.g., gavage, i.p. injection, vapor chambers, etc.; Sprow and Thiele, 2012). Factors like stress and alternative routes of administration may promote patterns of neurochemical expression that are distinct from alcohol ingestion alone. Therefore, it is not surprising that the results of this study yielded both similarities and differences from past work into brain monoaminergic responses to high levels of ethanol administration. Similar to previous work, ethanol access during DID increased measures reflecting striatal and brainstem area dopaminergic and serotonergic activity (see Figures 3, 5), which contain the mesolimbic DA system that is suggested to play a key role in the rewarding properties of alcohol (Koob, 2014; You et al., 2019). However, in contrast to past work, ethanol access produced relatively few neurochemical changes in the prefrontal cortex (see Figure 9), a brain region that has received some attention for monoaminergic responses following ethanol exposure (Fitzgerald, 2013). Moreover, alcohol access during DID had a relatively subtle influence on NE concentrations across brain areas. This is interesting because NE has been another focal point of past work investigating the acute and chronic effects of alcohol abuse (Koob and Kreek, 2007; Fitzgerald, 2013; Vazey et al., 2018). The reasons for distinctions between the current and past findings are not entirely clear. However, both the noradrenergic system and prefrontal cortex are key components of the stress response, which is more likely to be evoked during forced ethanol exposure (Koob, 2014; Vazey et al., 2018). Moreover, while mice ingested ethanol at levels comparable to the National Institute on Alcohol Abuse and Alcoholism standard for binge drinking (see Figure 2), studies employing techniques like gavage or i.p. injection more rapidly deliver greater volumes of ethanol (Rhodes et al., 2005, 2007; Thiele and Navarro, 2014). Therefore, perhaps exposure to even greater volumes of ethanol at faster rates or longer periods of alcohol exposure are required to evoke NE systems or monoamine responses in the prefrontal cortex similar to past work. Finally, the possibility remains that the estrus cycle of female mice in our study could have created variability in neurochemical concentrations, thereby masking the true magnitude of the effect sizes for the dependent measures. Such an outcome could contribute to deviations from the findings of past work. Despite possible limitations, these data underscore the utility of the DID model in adding a more comprehensive understanding of neurophysiological responses to acute alcohol abuse.

In conclusion, the results of the current study suggest that regularly engaging in moderate physical activity does not influence voluntary binge-patterned ethanol drinking in female mice, and may only mildly influence monoamine-related neurochemical responses to alcohol across brain areas that comprise motor, limbic, and reward circuits. Indeed, alcohol-related changes to monoaminergic levels in these brain areas, observed in the current study and others, have been suggested to contribute to the rewarding properties of alcohol, as well as cognitive dysfunction following episodes of alcohol abuse (Koob and Volkow, 2010). In summary, this work adds to a growing body of research that questions the utility of exercise as a therapeutic approach to curb incidences of alcohol misuse (Giesen et al., 2015; Leasure et al., 2015; Manthou et al., 2016), despite mounting evidence suggesting that regularly engaging in exercise may be more effective at reducing the risk for abusing other illicit substances (Goodwin, 2003; Smith and Lynch, 2012; Lynch et al., 2013).

## Data Availability Statement

The original contributions presented in the study are included in the article/Supplementary Material, further inquiries can be directed to the corresponding author.

## Ethics Statement

The animal study was reviewed and approved by Iowa State University Institutional Animal Care and Use Committee.

## Author Contributions

TB contributed to conceptualizing the study, performing experiments, and writing the manuscript. CR contributed to performing experiments and writing the manuscript. AS and EB contributed to performing experiments and data collection. RV contributed to performing experiments, training co-authors, and data collection. PC contributed to conceptualizing the study, training coauthors, writing the manuscript, and funding the entire study. All authors contributed to the article and approved the submitted version.

## Conflict of Interest

The authors declare that the research was conducted in the absence of any commercial or financial relationships that could be construed as a potential conflict of interest.
