# Corrigendum: The Influence of Moderate Physical Activity on Brain Monoaminergic Responses to Binge-Patterned Alcohol Ingestion in Female Mice

**DOI:** 10.3389/fnbeh.2021.780189

**Published:** 2021-10-25

**Authors:** Trevor J. Buhr, Carter H. Reed, Allyse Shoeman, Ella E. Bauer, Rudy J. Valentine, Peter J. Clark

**Affiliations:** ^1^Department of Food Science and Human Nutrition, Iowa State University, Ames, IA, United States; ^2^Neuroscience Program, Iowa State University, Ames, IA, United States; ^3^Interdepartmental Graduate Program in Nutritional Sciences, Iowa State University, Ames, IA, United States; ^4^Department of Kinesiology, Iowa State University, Ames, IA, United States

**Keywords:** alcohol abuse, drinking in the dark, exercise, physical activity, monoamine, rodent models, binge drinking, voluntary ethanol consumption

In the original article, there was a mistake in the legend for Figure 5 as published. The legend for Figure 5 does not contain the appropriate key to denote statistically significant differences detected by pairwise comparisons to match the text that corresponds to Figure 5. In: ‘dP < 0.05 Sed/Alcohol from Run/Water’, the superscript should be “d” rather than “b”. The correct legend appears below.

“**Figure 5**. Neurochemical levels in the mouse brainstem area immediately following the final DID session. The ratio of (HVA + DOPAC)/DA **(A)**, the ratio of 5-HIAA/5-HT **(B)**, L-DOPA concentrations **(C)**, 5-HIAA concentrations **(D)**, and 5-HT concentrations **(E)**. Data represent means +SEM. *P < 0.05 main effect alcohol access, ^∧^*P* < 0.05 main effect physical activity status, ^a^*P* < 0.05 Sed/Water from Sed/Alcohol, ^d^*P* < 0.05 Sed/Alcohol from Run/Water, ^e^*P* < 0.05 Sed/Alcohol from Run/Alcohol.”

In the original article, there was also a mistake in the legend for Figure 9 as published. The legend for Figure 9 does not contain the appropriate key to denote a statistically significant effect of alcohol access and differences detected by pairwise comparisons to match the text that corresponds to Figure 9. The correct legend appears below.

“**Figure 9**. Neurochemical levels in the mouse prefrontal cortex area immediately following the final DID session. NE concentrations **(A)**, and 5-HIAA concentrations **(B)**. Data represent means +SEM. *P < 0.05 main effect alcohol access, ^c^*P* < 0.05 Sed/Water from Run/Alcohol, ^f^*P* < 0.05 Run/Water from Run/Alcohol.”

The following figures also required corrections and have been replaced.

In the original article, there were mistakes in Figures 2, 5, 7, 8 and 9 as published.

In Figure 2B, the g/kg Ethanol that was reported represents only the last 2.5 h of the drinking in the dark paradigm, and not the total 4 h as stated in the legend. The amounts should have been reported as the total 4 h of ethanol access. The corrected Figure 2 appears below.

In Figure 5C, pairwise comparisons were labeled when the label to denote a significant main effect should have been used. This is correctly reported in the results section as a main effect but is incorrectly labeled in the corresponding Figure 5C. In Figure 5D, the “b” denoting a significant pairwise comparison should be changed to a “d” to accurately reflect the compared groups. This is correctly reported in the results section, just not the corresponding figure. The corrected Figure 5 appears below.

In Figure 7, the Figure key was not present. The pairwise comparison label, “c” in Figure 7C should be removed, as this group comparison was not statistically different. This was correctly described in the results section, but incorrectly labeled in the corresponding Figure 7C. The corrected Figure 7 appears below.

In Figure 8B, the significant pairwise comparison labels, “d” and “f” should be removed from above the “Run/Water” group as these were incorrectly included here but were correctly reported in the corresponding text of the results section. The corrected Figure 8 appears below.

In Figure 9A, the pairwise comparison label, “d” should be removed as this group comparison was not statistically different. This was indicated in the text correctly, but incorrectly labeled in the corresponding Figure 9A. In Figure 9B, pairwise comparisons were labeled when notation to denote a significant main effect should have been used. This is correctly reported in the results section as a main effect but is incorrectly labeled in the Figure 9B. The corrected Figure 9 appears below.

Lastly, in the original article, there was an error in the “Drinking Data” paragraph of the Results section. The amount of g/kg ethanol consumption that was reported only reflected the last 2.5 h of the drinking in the dark paradigm rather than the entire 4-h period.

A correction has been made to *Results, Drinking Data*. The corrected paragraph is shown below.

“Physical activity status did not influence the amount of fluid ingested relative to bodyweight for either mice that had access to water or alcohol (*F*_(1,32)_ = 0.00, *p* = 0.94; see Figure 2A). However, mice with access to ethanol consumed greater volumes of fluid compared to those with access to water during DID (*F*_(3,32)_ = 7.293, *p* < 0.0007). Mice consumed almost double the volume of ethanol or water on day 5 during the 4-h DID session, when compared to the first 4 days that consisted of 2-h DID sessions (*F*_(4,123)_ = 32.26, *p* < 0.0001; see Figure 2A). The amount of ethanol ingested on day 5 of DID was 8.82 g/kg (±SEM = 0.8) for sedentary mice and 9.36 g/kg (±SEM = 0.4) for mice with access to running wheels (see Figure 2B). Finally, wheel access did not influence the amounts of fluids ingested independent of fluid type (*F*_(1,32)_ = 2.87, *p* = 0.11), or while considering day and fluid type (*F*_(4,123)_ = 0.52, *p* = 0.72).”

**Figure 2:**
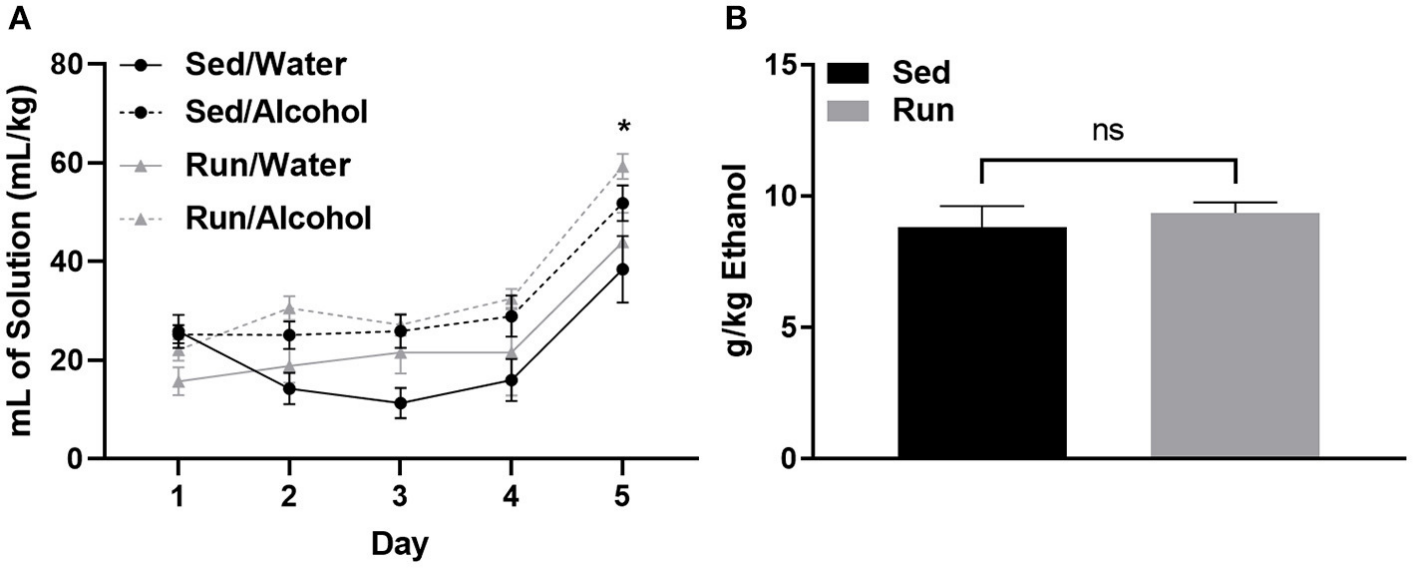
Ethanol consumption during the drinking in the dark protocol. Comparison between the average amount of fluid ingested (±SEM) over the 5-day DID procedure (relative to body mass) for mice that had access to alcohol and water **(A)** and total ethanol intake (relative to body mass) during the final DID session **(B)**. *P < 0.05 Main effect of day, ^ns^*P* > 0.05 between Run/Alcohol and Sed/Alcohol. Data represent means ±SEM.

**Figure 5:**
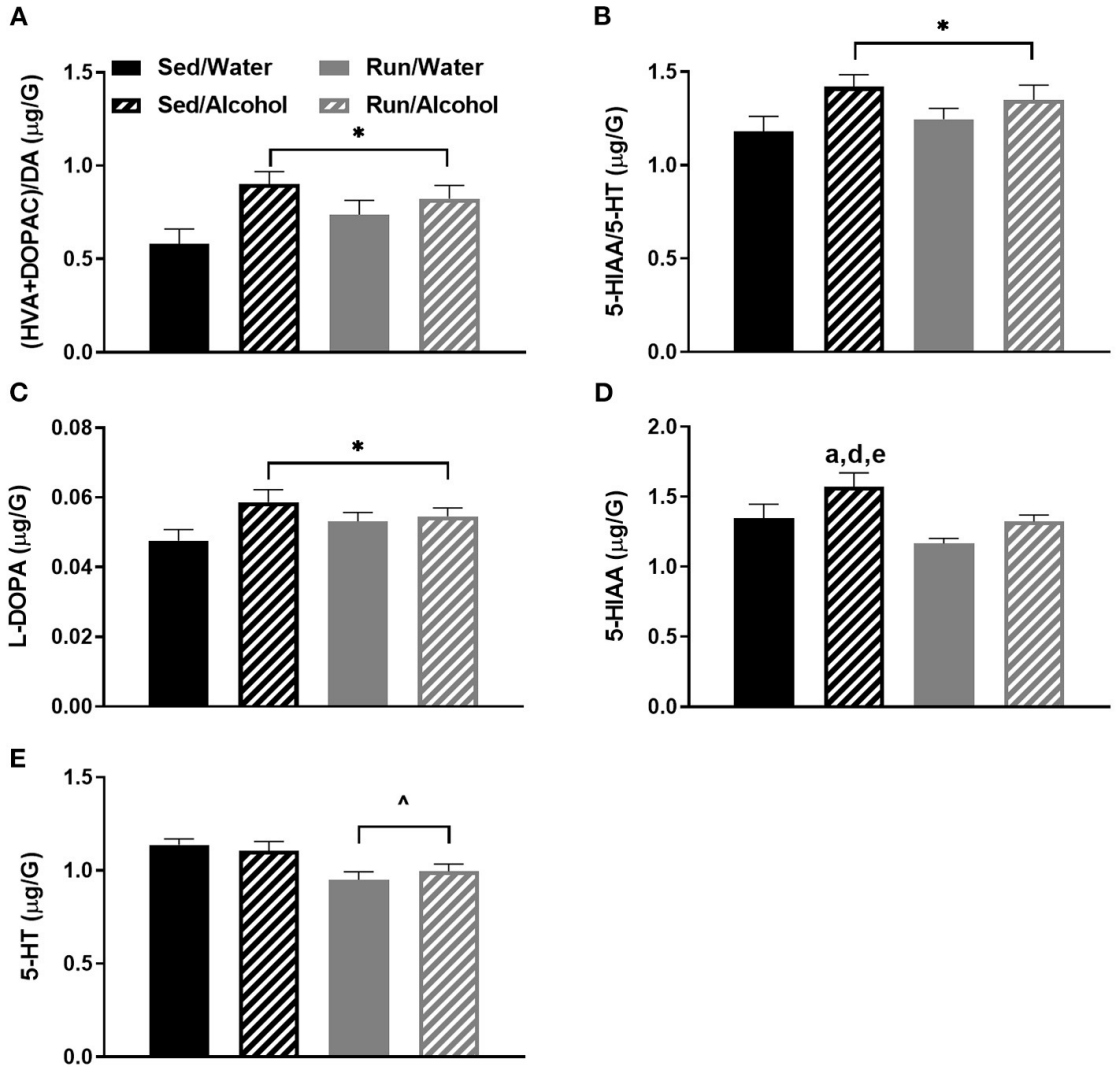
Neurochemical levels in the mouse brainstem area immediately following the final DID session. The ratio of (HVA + DOPAC)/DA **(A)**, the ratio of 5-HIAA/5-HT **(B)**, L-DOPA concentrations **(C)**, 5-HIAA concentrations **(D)**, and 5-HT concentrations **(E)**. Data represent means +SEM. *P < 0.05 main effect alcohol access, ^∧^*P* < 0.05 main effect physical activity status, ^a^*P* < 0.05 Sed/Water from Sed/Alcohol, ^d^*P* < 0.05 Sed/Alcohol from Run/Water, ^e^P < 0.05 Sed/Alcohol from Run/Alcohol.

**Figure 7:**
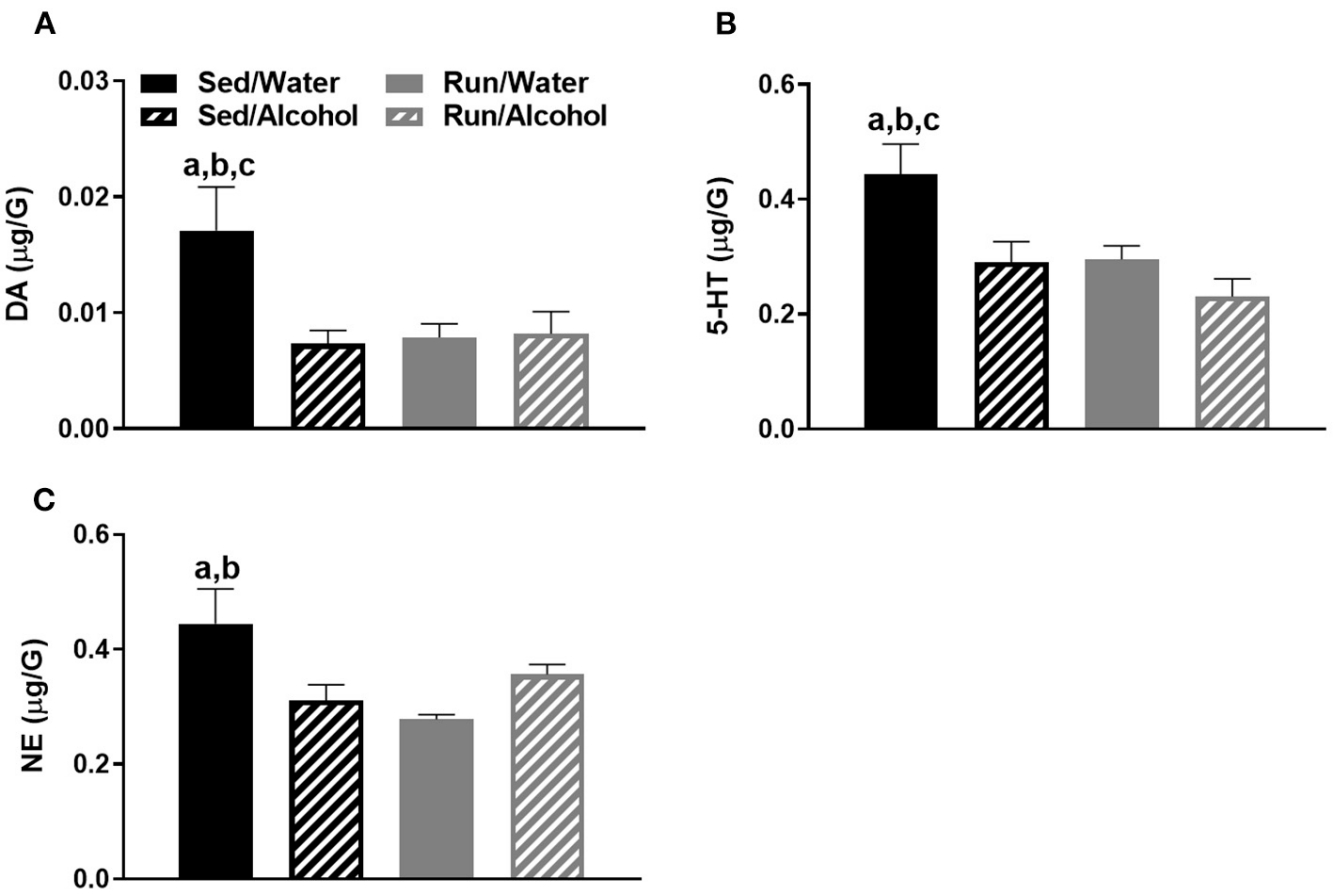
Neurochemical levels in the mouse cerebellum area immediately following the final DID session. DA concentrations **(A)**, 5-HT concentrations **(B)**, and NE concentrations **(C)**. Data represent means +SEM. ^a^*P* < 0.05 Sed/Water from Sed/Alcohol, ^b^*P* < 0.05 Sed/Water from Run/Water, ^c^*P* < 0.05 Sed/Water from Run/Alcohol.

**Figure 8:**
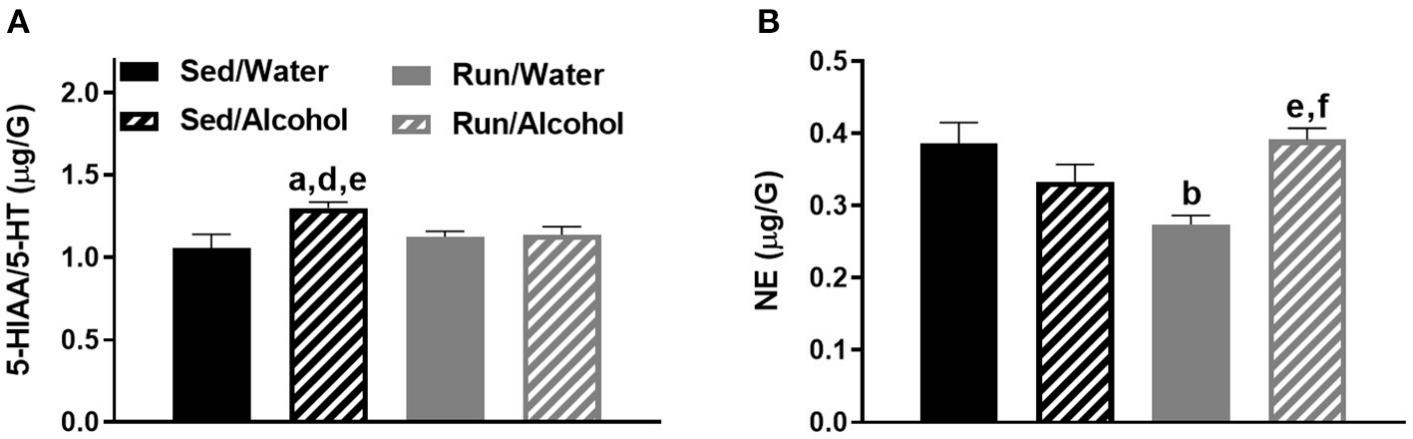
Neurochemical levels in the mouse hippocampus area immediately following the final DID session. The ratio of 5-HIAA/5-HT **(A)**, and NE concentrations **(B)**. Data represent means +SEM. ^a^*P* < 0.05 Sed/Water from Sed/Alcohol, ^b^*P* < 0.05 Sed/Water from Run/Water, ^d^*P* < 0.05 Sed/Alcohol from Run/Water, ^e^*P* < 0.05 Sed/Alcohol from Run/Alcohol, ^f^*P* < 0.05 Run/Water from Run/Alcohol.

**Figure 9:**
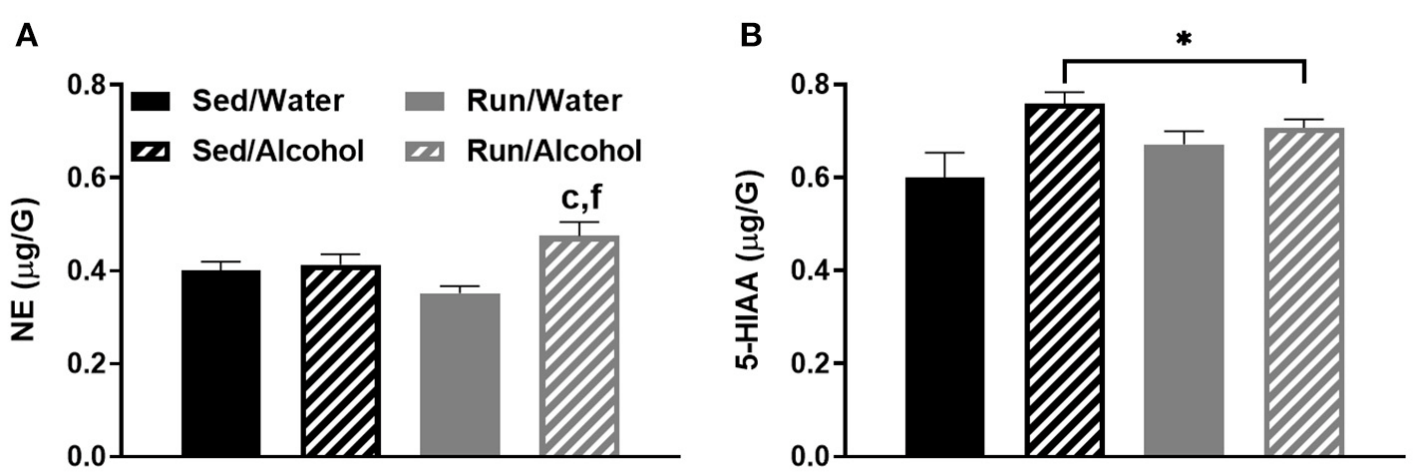
Neurochemical levels in the mouse prefrontal cortex area immediately following the final DID session. NE concentrations **(A)**, and 5-HIAA concentrations **(B)**. Data represent means +SEM. *P < 0.05 main effect alcohol access, ^c^*P* < 0.05 Sed/Water from Run/Alcohol, ^f^*P* < 0.05 Run/Water from Run/Alcohol.

The authors apologize for these errors and state that they do not change the scientific conclusions of the article in any way. The original article has been updated.

## Publisher's Note

All claims expressed in this article are solely those of the authors and do not necessarily represent those of their affiliated organizations, or those of the publisher, the editors and the reviewers. Any product that may be evaluated in this article, or claim that may be made by its manufacturer, is not guaranteed or endorsed by the publisher.

