# Supplementary material for: The Influence of Moderate Physical Activity on Brain Monoaminergic Responses to Binge-Patterned Alcohol Ingestion in Female Mice
Source: Front Behav Neurosci. 2021 Feb 25;15:639790. doi: 10.3389/fnbeh.2021.639790 (PMC7947191; doi:10.3389/fnbeh.2021.639790)
Supplement: Supplementary file 1 [file Data_Sheet_1.PDF]

Supplementary Material

Supplementary Table 1.1

All Mean L-DOPA Concentrations (µg/G).

Treatment Condition	Sed/Water			Sed/Alcohol			Run/Water			Run/Alcohol			Two-Way ANOVA Statistics		
**Brain Area**	**Mean**	**SEM**	**N**	**Mean**	**SEM**	**N**	**Mean**	**SEM**	**N**	**Mean**	**SEM**	**N**	**Exercise Effect**	**Alcohol Effect**	**Interaction**
Striatum area	0.06229	0.00457	7	0.06013	0.00404	8	0.053	0.00679	8	0.07411	0.00235	9	F(1,32)=0.25, *p*=0.62	F(1,32)=4.30, *p*=0.04	F(1,32)=6.30, *p*=0.01
Hypothalamus area	0.23167	0.01507	9	0.24878	0.02065	9	0.222	0.01193	7	0.25922	0.02041	9	F(1,32)=0.00, *p*=0.98	F(1,32)=2.23, *p*=0.14	F(1,32)=0.31, *p*=0.58
Brainstem area	0.04756	0.00325	9	0.05878	0.00361	9	0.053	0.00252	9	0.05456	0.00236	9	F(1,32)=0.06, *p*=0.08	F(1,32)=4.47, *p*=0.04	F(1,32)=2.65, *p*=0.11
Caudal cortical area	0.01567	0.00496	6	0.04444	0.0142	9	0.02467	0.00633	9	0.02967	0.00519	9	F(1,32)=0.10, *p*=0.75	F(1,32)=3.23, *p*=0.08	F(1,32)=1.63, *p*=0.21
Cerebellum area	0.06278	0.01128	9	0.06511	0.00399	9	0.06167	0.00213	9	0.05878	0.00417	9	F(1,32)=0.34, *p*=0.56	F(1,32)=0.00, *p*=0.95	F(1,32)=0.17, *p*=0.68
Hippocampus area	0.01725	0.00475	8	0.022	0.00264	9	0.01578	0.00191	9	0.02175	0.00258	8	F(1,32)=0.36, *p*=0.55	F(1,32)=1.61, *p*=0.21	F(1,32)=0.07, *p*=0.79
Prefrontal cortex area	0.07775	0.0067	8	0.09233	0.00338	9	0.097	0.00496	9	0.08656	0.0118	9	F(1,32)=0.07, *p*=0.78	F(1,32)=0.82, *p*=0.37	F(1,32)=2.86, *p*=0.10

Supplementary Table 1.2

All Mean DA Concentrations (μg/G).

Treatment Condition	Sed/Water			Sed/Alcohol			Run/Water			Run/Alcohol			Two-Way ANOVA Statistics		
**Brain Area**	**Mean**	**SEM**	**N**	**Mean**	**SEM**	**N**	**Mean**	**SEM**	**N**	**Mean**	**SEM**	**N**	**Exercise Effect**	**Alcohol Effect**	**Interaction**
Striatum area	4.86633	0.16555	9	4.36089	0.15701	9	4.41356	0.13539	9	4.66856	0.19549	9	F(1,32)=0.19, *p*=0.66	F(1,32)=0.58, *p*=0.45	F(1,32)=5.33, *p*=0.02
Hypothalamus area	0.48911	0.02872	9	0.50211	0.04628	9	0.42533	0.01791	9	0.53933	0.04816	9	F(1,32)=0.13, *p*=0.72	F(1,32)=2.88, *p*=0.09	F(1,32)=1.80, *p*=0.18
Brainstem area	0.148	0.03837	9	0.08322	0.00483	9	0.08067	0.0124	9	0.08956	0.01555	9	F(1,32)=1.95, *p*=0.17	F(1,32)=1.65, *p*=0.20	F(1,32)=2.86, *p*=0.10
Caudal cortical area	0.38922	0.0351	9	0.48511	0.06843	9	0.48378	0.05083	9	0.59378	0.11336	9	F(1,32)=1.94, *p*=0.17	F(1,32)=1.98, *p*=0.16	F(1,32)=0.01, *p*=0.92
Cerebellum area	0.01711	0.00376	9	0.00733	0.00116	9	0.00789	0.00118	9	0.00822	0.00189	9	F(1,32)=3.69, p=0.06	F(1,32)=4.45, *p*=0.04	F(1,32)=4.69, p=0.03
Hippocampus area	0.017	0.00564	9	0.00889	0.00145	9	0.026	0.01742	9	0.01433	0.00385	9	F(1,32)=0.56, *p*=0.46	F(1,32)=1.11, *p*=0.29	F(1,32)=0.04, *p*=0.84
Prefrontal cortex area	0.13244	0.04623	9	0.18878	0.08678	9	0.09778	0.02211	9	0.18633	0.06772	9	F(1,32)=1.05, *p*=0.31	F(1,32)=0.10, *p*=0.75	F(1,32)=1.01, *p*=0.32

Supplementary Table 1.3

All Mean HVA Concentrations (μg/G).

Treatment Condition	Sed/Water			Sed/Alcohol			Run/Water			Run/Alcohol			Two-Way ANOVA Statistics		
**Brain Area**	**Mean**	**SEM**	**N**	**Mean**	**SEM**	**N**	**Mean**	**SEM**	**N**	**Mean**	**SEM**	**N**	**Exercise Effect**	**Alcohol Effect**	**Interaction**
Striatum area	0.31333	0.01626	9	0.36256	0.01798	9	0.30989	0.00985	9	0.39967	0.02732	9	F(1,32)=0.78, p=0.38	F(1,32)=13.53, p=0.0009	F(1,32)=1.15, p=0.29
Hypothalamus area	0.16778	0.00826	9	0.18144	0.01681	9	0.19656	0.0262	9	0.20922	0.01656	9	F(1,32)=2.47, p=0.12	F(1,32)=0.53, p=0.47	F(1,32)=0.00, p=0.97
Brainstem area	0.04456	0.00467	9	0.04778	0.00352	9	0.03544	0.00237	9	0.04367	0.00319	9	F(1,32)=3.55, p=0.06	F(1,32)=2.76, p=0.10	F(1,32)=0.44, p=0.51
Caudal cortical area	0.04322	0.00369	9	0.06489	0.00455	9	0.05111	0.00334	9	0.06967	0.00962	9	F(1,32)=1.18, p=0.28	F(1,32)=11.75, p=0.001	F(1,32)=0.05, p=0.82
Cerebellum area	0.00511	0.00203	9	0.00222	0.00078	9	0.00144	0.00044	9	0.00144	0.00067	9	F(1,32)=3.53, p=0.06	F(1,32)=1.95, p=0.17	F(1,32)=1.35, p=0.25
Hippocampus area	0.02122	0.00917	9	0.01411	0.00089	9	0.01511	0.00285	9	0.02511	0.00669	9	F(1,32)=0.18, p=0.67	F(1,32)=0.05, p=0.82	F(1,32)=2.08, p=0.15
Prefrontal cortex area	0.10878	0.01187	9	0.11233	0.00744	9	0.08722	0.00322	9	0.12889	0.01997	9	F(1,32)=0.04, p=0.84	F(1,32)=3.39, p=0.07	F(1,32)=2.41, p=0.13

Supplementary Table 1.4

All Mean DOPAC Concentrations (µg/G).

Treatment Condition	Sed/Water			Sed/Alcohol			Run/Water			Run/Alcohol			Two-Way ANOVA Statistics		
**Brain Area**	**Mean**	**SEM**	**N**	**Mean**	**SEM**	**N**	**Mean**	**SEM**	**N**	**Mean**	**SEM**	**N**	**Exercise Effect**	**Alcohol Effect**	**Interaction**
Striatum area	0.34189	0.02144	9	0.38122	0.02964	9	0.337	0.02184	9	0.42922	0.04766	9	F(1,32)=0.45, *p*=0.50	F(1,32)=4.22, *p*=0.04	F(1,32)=0.68, *p*=0.41
Hypothalamus area	0.11622	0.00616	9	0.12411	0.00683	9	0.11467	0.00932	9	0.14533	0.01201	9	F(1,32)=1.23, *p*=0.27	F(1,32)=4.75, *p*=0.03	F(1,32)=1.61, *p*=0.21
Brainstem area	0.02411	0.00262	9	0.02611	0.0022	9	0.01878	0.00252	9	0.02278	0.00221	9	F(1,32)=3.24, *p*=0.08	F(1,32)=1.67, *p*=0.20	F(1,32)=0.15, *p*=0.70
Caudal cortical area	0.02578	0.00341	9	0.039	0.00431	9	0.03244	0.00288	9	0.04389	0.00702	9	F(1,32)=1.48, *p*=0.23	F(1,32)=6.99, *p*=0.01	F(1,32)=0.04, *p*=0.84
Cerebellum area	0.00822	0.00301	9	0.00356	0.00087	9	0.00244	0.00094	9	0.00267	0.00073	9	F(1,32)=3.86, *p*=0.06	F(1,32)=1.74, *p*=0.19	F(1,32)=1.97, *p*=0.17
Hippocampus area	0.00189	0.00189	9	0	0	9	0.00044	0.00044	9	0.00144	0.00144	9	F(1,32)=0.00, *p*=0.97	F(1,32)=0.13, *p*=0.72	F(1,32)=1.48, *p*=0.23
Prefrontal cortex area	0.05622	0.01834	9	0.05422	0.01049	9	0.03333	0.00433	9	0.05033	0.01028	9	F(1,32)=1.23, *p*=0.27	F(1,32)=0.41, *p*=0.52	F(1,32)=0.64, *p*=0.42

Supplementary Table 1.5

All Mean (HVA+DOPAC)/DA Concentrations (µg/G).

Treatment Condition	Sed/Water			Sed/Alcohol			Run/Water			Run/Alcohol			Two-Way ANOVA Statistics		
**Brain Area**	**Mean**	**SEM**	**N**	**Mean**	**SEM**	**N**	**Mean**	**SEM**	**N**	**Mean**	**SEM**	**N**	**Exercise Effect**	**Alcohol Effect**	**Interaction**
Striatum area	0.13456	0.00524	9	0.17089	0.00901	9	0.14644	0.00474	9	0.18056	0.01926	9	F(1,32)=9.86, *p*=0.003	F(1,32)=0.94, *p*=0.34	F(1,32)=0.01, *p*=0.91
Hypothalamus area	0.59233	0.03618	9	0.62644	0.03577	9	0.735	0.05602	9	0.672	0.04288	9	F(1,32)=0.11, *p*=0.74	F(1,32)=4.68, *p*=0.03	F(1,32)=1.24, *p*=0.27
Brainstem area	0.582	0.07892	9	0.90156	0.06665	9	0.73811	0.07626	9	0.82322	0.07129	9	F(1,32)=7.59, *p*=0.009	F(1,32)=0.28, *p*=0.60	F(1,32)=2.55, *p*=0.12
Caudal cortical area	0.17922	0.01393	9	0.22878	0.01642	9	0.18133	0.01319	9	0.22211	0.02544	9	F(1,32)=6.31, *p*=0.01	F(1,32)=0.01, *p*=0.90	F(1,32)=0.06, *p*=0.80
Cerebellum area	0.63678	0.16532	9	0.63778	0.15784	9	0.47878	0.13534	9	0.45544	0.07611	9	F(1,32)=0.01, *p*=0.93	F(1,32)=1.52, *p*=0.22	F(1,32)=0.01, *p*=0.92
Hippocampus area	1.15822	0.11511	9	1.85578	0.26905	9	1.659	0.34618	9	2.328	0.55093	9	F(1,32)=3.67, *p*=0.06	F(1,32)=1.86, *p*=0.18	F(1,32)=0.00, *p*=0.96
Prefrontal cortex area	1.64656	0.2383	9	1.61389	0.28537	9	1.477	0.15099	9	1.728	0.39063	9	F(1,32)=0.15, *p*=0.69	F(1,32)=0.01, *p*=0.92	F(1,32)=0.26, *p*=0.61

Supplementary Table 1.6

All Mean 5-HT Concentrations (μg/G).

Treatment Condition	Sed/Water			Sed/Alcohol			Run/Water			Run/Alcohol			Two-Way ANOVA Statistics		
**Brain Area**	**Mean**	**SEM**	**N**	**Mean**	**SEM**	**N**	**Mean**	**SEM**	**N**	**Mean**	**SEM**	**N**	**Exercise Effect**	**Alcohol Effect**	**Interaction**
Striatum area	1.05389	0.03197	9	0.92944	0.03976	9	0.97044	0.02332	9	0.97622	0.05608	9	F(1,32)=0.22, p=0.64	F(1,32)=2.23, p=0.14	F(1,32)=2.70, p=0.11
Hypothalamus area	2.75656	0.17181	9	2.51944	0.1596	9	1.925	0.18894	9	2.84256	0.27675	9	F(1,32)=1.54, p=0.22	F(1,32)=2.77, p=0.10	F(1,32)=7.97, p=0.008
Brainstem area	1.13744	0.03185	9	1.10622	0.04923	9	0.95056	0.04209	9	0.99511	0.03879	9	F(1,32)=13.25, p=0.001	F(1,32)=0.03, p=0.87	F(1,32)=0.86, p=0.36
Caudal cortical area	0.49022	0.02498	9	0.47644	0.02633	9	0.45544	0.02125	9	0.51622	0.04096	9	F(1,32)=0.01, p=0.92	F(1,32)=0.64, p=0.43	F(1,32)=1.62, p=0.21
Cerebellum area	0.44411	0.05205	9	0.29089	0.03557	9	0.29533	0.02368	9	0.23056	0.03108	9	F(1,32)=5.90, p=0.02	F(1,32)=6.22, p=0.01	F(1,32)=2.35, p=0.13
Hippocampus area	1.07689	0.08106	9	1.05	0.03093	9	1.00133	0.05873	9	1.08644	0.09576	9	F(1,32)=0.08, p=0.78	F(1,32)=0.17, p=0.68	F(1,32)=0.62, p=0.43
Prefrontal cortex area	0.92789	0.05857	9	0.97278	0.02805	9	0.90811	0.01622	9	0.94	0.07511	9	F(1,32)=0.27, p=0.60	F(1,32)=0.58, p=0.45	F(1,32)=0.02, p=0.89

Supplementary Table 1.7

All Mean 5-HIAA Concentrations (µg/G).

Treatment Condition	Sed/Water			Sed/Alcohol			Run/Water			Run/Alcohol			Two-Way ANOVA Statistics		
**Brain Area**	**Mean**	**SEM**	**N**	**Mean**	**SEM**	**N**	**Mean**	**SEM**	**N**	**Mean**	**SEM**	**N**	**Exercise Effect**	**Alcohol Effect**	**Interaction**
Striatum area	0.72944	0.03969	9	0.75689	0.01881	9	0.68411	0.01943	9	0.75533	0.03403	9	F(1,32)=0.64, p=0.42	F(1,32)=2.80, p=0.10	F(1,32)=0.55, p=0.46
Hypothalamus area	2.30967	0.11587	9	2.44178	0.1883	9	1.74978	0.21173	9	2.50522	0.15282	9	F(1,32)=3.32, p=0.07	F(1,32)=6.73, p=0.01	F(1,32)=2.11, p=0.15
Brainstem area	1.34622	0.10066	9	1.57189	0.09881	9	1.168	0.03479	9	1.32378	0.0459	9	F(1,32)=7.82, p=0.008	F(1,32)=6.27, p=0.01	F(1,32)=0.21, p=0.64
Caudal cortical area	0.409	0.03249	9	0.44844	0.02436	9	0.39389	0.02178	9	0.45378	0.0224	9	F(1,32)=0.04, p=0.84	F(1,32)=3.75, p=0.06	F(1,32)=0.15, p=0.69
Cerebellum area	0.64411	0.14069	9	0.39544	0.04027	9	0.35244	0.01849	9	0.37556	0.01523	9	F(1,32)=2.31, p=0.13	F(1,32)=4.41, p=0.04	F(1,32)=3.36, p=0.07
Hippocampus area	1.16044	0.14207	9	1.36978	0.06861	9	1.11844	0.05908	9	1.20811	0.08329	9	F(1,32)=1.18, p=0.28	F(1,32)=2.53, p=0.12	F(1,32)=0.41, p=0.52
Prefrontal cortex area	0.59978	0.0538	9	0.759	0.02442	9	0.67144	0.0284	9	0.70722	0.01784	9	F(1,32)=0.09, p=0.77	F(1,32)=8.21, p=0.007	F(1,32)=3.29, p=0.07

Supplementary Table 1.8

All Mean 5-HIAA/5-HT Concentrations (μg/G).

Treatment Condition	Sed/Water			Sed/Alcohol			Run/Water			Run/Alcohol			Two-Way ANOVA Statistics		
**Brain Area**	**Mean**	**SEM**	**N**	**Mean**	**SEM**	**N**	**Mean**	**SEM**	**N**	**Mean**	**SEM**	**N**	**Exercise Effect**	**Alcohol Effect**	**Interaction**
Striatum area	0.69372	0.03611	9	0.82476	0.03734	9	0.70671	0.01985	9	0.79401	0.06087	9	$F(1,32)=0.05$, $p=0.83$	$F(1,32)=7.01$, $p=0.01$	$F(1,32)=0.28$, $p=0.59$
Hypothalamus area	0.85625	0.05299	9	0.9715	0.04154	9	0.88904	0.03793	9	0.9177	0.06008	9	$F(1,32)=0.05$, $p=0.83$	$F(1,32)=2.16$, $p=0.15$	$F(1,32)=0.78$, $p=0.38$
Brainstem area	1.18139	0.07914	9	1.41995	0.06485	9	1.24551	0.05939	9	1.34944	0.07906	9	$F(1,32)=0.00$, $p=0.96$	$F(1,32)=5.79$, $p=0.02$	$F(1,32)=0.90$, $p=0.35$
Caudal cortical area	0.8341	0.04601	9	0.94635	0.03268	9	0.86592	0.03107	9	0.90342	0.05834	9	$F(1,32)=0.02$, $p=0.89$	$F(1,32)=2.97$, $p=0.09$	$F(1,32)=0.74$, $p=0.39$
Cerebellum area	1.49012	0.342	9	1.3901	0.06305	9	1.21664	0.04895	9	1.46175	0.08825	8	$F(1,32)=0.30$, $p=0.59$	$F(1,32)=0.15$, $p=0.69$	$F(1,32)=0.86$, $p=0.35$
Hippocampus area	1.05502	0.08418	9	1.2993	0.03708	9	1.12469	0.03328	9	1.13722	0.04823	9	$F(1,32)=0.72$, $p=0.40$	$F(1,32)=5.54$, $p=0.02$	$F(1,32)=4.51$, $p=0.04$
Prefrontal cortex area	0.63954	0.03687	9	0.78367	0.0285	9	0.73939	0.02798	9	0.80655	0.09395	9	$F(1,32)=1.28$, $p=0.26$	$F(1,32)=3.79$, $p=0.06$	$F(1,32)=0.50$, $p=0.48$

Supplementary Table 1.9

All Mean NE Concentrations (µg/G).

Treatment Condition	Sed/Water			Sed/Alcohol			Run/Water			Run/Alcohol			Two-Way ANOVA Statistics		
**Brain Area**	**Mean**	**SEM**	**N**	**Mean**	**SEM**	**N**	**Mean**	**SEM**	**N**	**Mean**	**SEM**	**N**	**Exercise Effect**	**Alcohol Effect**	**Interaction**
Striatum area	0.39089	0.00768	9	0.36956	0.01096	9	0.34344	0.01472	9	0.40922	0.01773	9	$F(1,32)=0.09$, $p=0.77$	$F(1,32)=2.79$, $p=0.10$	$F(1,32)=10.7$, $p=0.002$
Hypothalamus area	1.73656	0.09665	9	1.77967	0.15899	9	1.36967	0.05085	9	1.97389	0.18475	9	$F(1,32)=0.42$, $p=0.52$	$F(1,32)=5.87$, $p=0.02$	$F(1,32)=4.42$, $p=0.04$
Brainstem area	0.54289	0.01864	9	0.55889	0.02093	9	0.50556	0.01749	9	0.54411	0.01593	9	$F(1,32)=2.00$, $p=0.16$	$F(1,32)=2.19$, $p=0.14$	$F(1,32)=0.39$, $p=0.53$
Caudal cortical area	0.29722	0.01296	9	0.27767	0.01089	9	0.26478	0.0072	9	0.30689	0.01225	9	$F(1,32)=0.03$, $p=0.87$	$F(1,32)=1.02$, $p=0.31$	$F(1,32)=7.78$, $p=0.008$
Cerebellum area	0.44489	0.06014	9	0.31111	0.02734	9	0.27844	0.00786	9	0.35622	0.01755	9	$F(1,32)=3.12$, $p=0.08$	$F(1,32)=0.66$, $p=0.42$	$F(1,32)=9.49$, $p<0.05$
Hippocampus area	0.38667	0.02836	9	0.33311	0.02404	9	0.27378	0.01248	9	0.396	0.01761	9	$F(1,32)=1.34$, $p=0.25$	$F(1,32)=2.55$, $p=0.12$	$F(1,32)=16.69$, $p=0.0003$
Prefrontal cortex area	0.4	0.01969	9	0.41311	0.02229	9	0.35178	0.01531	9	0.47522	0.03001	9	$F(1,32)=0.10$, $p=0.75$	$F(1,32)=9.20$, $p=0.004$	$F(1,32)=6.06$, $p=0.01$

Supplementary Table 2.1

Mean Brain Sample Weight (g).

Brain Area	Mean	SEM	N
Striatum area	0.0713222	0.0016328	36
Hypothalamus area	0.0107667	0.0004867	36
Brainstem area	0.0981806	0.003387	36
Caudal Cortical area	0.1263028	0.0047036	36
Cerebellum area	0.052775	0.0017409	36
Hippocampus area	0.0385917	0.00099	36
Prefrontal Cortex area	0.0342642	0.0013972	36

frontiers
